# Supplementary material for: Relative Importance of Determinants of Changes in Eating Behavior during the Transition to Parenthood: Priorities for Future Research and Interventions
Source: Nutrients. 2021 Jul 15;13(7):2429. doi: 10.3390/nu13072429 (PMC8308599; doi:10.3390/nu13072429)
Supplement: Supplementary file 1 [file nutrients-13-02429-s001.zip › nutrients-1284921-supplementary.pdf]

Supplementary Tables S1–S6

Supplementary Table S1. Overview of determinants of changes in eating behavior during pregnancy, derived from Versele et al. [11].

Level / Sublevel	Determinant	Explanation
**Individual**		
**Biological**		
	**Cravings**	A powerful desire for specific foods, e.g., sweet foods.
	**Discomfort**	Physical unease, a painful/uncomfortable feeling in certain part(s) of the body. *Extra clarification for the experts regarding the rating for pregnant women: E.g., as a result of pregnancy, such as heartburn).*
	**Fatigue**	Extreme tiredness resulting from mental or physical exertion (or from pregnancy), resulting in e.g. being too tired to prepare food or to go for groceries.
	**Hunger and satiety**	Feeling hungry, sometimes in combination with not knowing which foods give a saturated feeling.
	**Physiological changes** *(rated only by/for women)*	Body changes (e.g., as a result of pregnancy). These changes were e.g. described as the feeling that their body is working hard to grow the baby, that it became physically difficult to cook, …
	**Sickness**	The feeling or fact of being affected with nausea or vomiting.
	**Specific food preferences**	Changing preference or aversion for certain foods. A greater (dis)liking for specific foods over another or other foods. *Extra clarification for the experts regarding the rating for pregnant women: some pregnant women no longer felt like eating some kinds of fruit, vegetables, dishes, etc. at the beginning/middle/end or throughout their pregnancy.*
	**Taste palette**	Changes in the sense of taste during pregnancy (of the partner).
	**Taste preference**	A change in the preference of flavor perceived in the mouth and throat. This can be expressed by e.g. more sweet, salty,… preferences.
**Psychological**		
	**Anticipation**	Preparing oneself, looking ahead at things. Such as having food around in case of becoming hungry.
	**Eating regulation**	Being able to control the eating pattern. Ability to maintain a healthy and balanced diet.
	**Food knowledge**	Facts, information, and acquaintance acquired through experience or education about food. Lack of information about what can be eaten and what not during pregnancy (of the partner).
	**Habits**	A settled or regular tendency or practice related to eating behavior, especially one that is hard to change or give up. *Extra clarification for the experts regarding the rating for pregnant women: Some pregnant women e.g. stated that new*

	behaviors as a result of their pregnancy (e.g. eating more in between meals to avoid nausea) become a new habit afterwards. *Extra clarification for the experts regarding the rating for expecting fathers: Some expecting fathers e.g. stated that new behaviors as a result of the pregnancy of their partner (e.g. eating along with pregnant partner) became a new habit afterwards.*
**Health concerns**	Own or baby's health related matter that is of interest, importance or worry to parents to be. E.g. being more aware of what one eats and what (health) consequences this has for the baby.
**Health consciousness**	Attitude in which one has awareness of the healthiness of one's diet and lifestyle.
**Mood and emotions**	A temporary state of mind or feeling deriving from one's circumstances. E.g. eating specific foods or at specific moments as a result of feelings and emotions such as boredom, stress.
**(Perceived) food safety**	Interpretation of and response to food safety issues related with pregnancy (of the partner) in a particular way. *Extra clarification for the experts regarding the rating for pregnant women: Some pregnant women e.g. did not dare to eat everything they wanted during pregnancy because of (perceived) safety issues.*
**Pregnancy card, self-licensing**	Allowing themselves more because they are pregnant/the partner is pregnant. *Extra clarification for the experts regarding the rating for pregnant women: E.g. some pregnant women described it as their 'pregnancy card'.*
**Self-control**	The ability to control oneself, in particular one's emotions and desires, especially in difficult situations, e.g. by trying not to give in to certain cravings.
**Self-efficacy**	One's belief in one's ability or capacity to succeed in specific situations or accomplish a task. E.g. some people stated that some things are out of their control and they cannot do anything about it.
**Weight control**	Paying attention to a healthy weight (gain). *Extra clarification for the experts regarding the rating for pregnant women: E.g. making sure weight gain is mainly due to the growing baby and related tissues (placenta, extra blood, …), and not because of paying less attention to what one eats.* *Extra clarification for the experts regarding the rating for expecting fathers: E.g. maintaining a healthy body weight throughout the pregnancy of the partner.*
**Worries and concerns**	Fears or concerns about actual or potential problems. *Extra clarification for the experts regarding the rating for pregnant women: E.g., uncertainties about body weight and food choices, fear for gaining too much weight, or fearing the results of the 'Glucose screening test' etc.*

		Extra clarification for the experts regarding the rating for pregnant women: E.g., uncertainties about body weight and food choices.
**Situational**		
	**Effort and convenience**	Presence or absence of a determined attempt related to eating behavior, the state of being (un)able to proceed with something without difficulty. This makes that some people e.g. do not feel like cooking or spending time on healthy meals, as it requires too much effort. This could thus result in the choice for more take away or unhealthy, convenient foods.
	**Other priorities**	To prioritize other activities over healthy cooking/eating. E.g. cooking is often not people's first priority; some people stated they want to enjoy free time as much as possible before the baby is born.
	**Time constraints**	Time limitations or restrictions. E.g. some people stated that cooking takes (too much) time.
**Interpersonal**		
	**Influence of female/male partner**	The effect or influence from the pregnant woman on the behavior of her non-pregnant partner (man)/ The effect or influence from the non-pregnant partner (man) on the behavior of the pregnant partner (woman).
	**Professional influence**	Nutritional advice and assistance from professionals. *Extra clarification for the experts regarding the rating for pregnant women: E.g. the gynecologist can give certain recommendations or warnings, such as a warning for gestational diabetes.*
	**Sensitivity to others' opinions**	Being sensitive to the opinions of other people. E.g. when others say that you should or should not eat certain foods.
	**Social pressure to eat**	A certain persuasion or intimidation from others to eat more. *Extra clarification for the experts regarding the rating for pregnant women: E.g. the assumption that pregnant women "have to eat for two".*
**Environmental**		
**Micro**		
	**Home food availability**	The amounts and types of food that are physically (un)available at home. E.g. when people do not buy (un)healthy foods.
**Meso/Macro**		
	**Environment food availability**	Accessibility, availability, and adequacy of food within a certain environment. E.g. some people mentioned that it can be difficult to find healthy alternatives for foods pregnant women cannot eat because of food safety issues in restaurants.

Supplementary Table S2. Overview of determinants of changes in eating behavior within the first year postpartum, derived from Versele et al. [11].

**Level** / **Sublevel**	**Determinant**	**Explanation**

**Individual**			
	**Biological**		
		**Discomfort**	Physical unease, a painful/uncomfortable feeling in certain part(s) of the body.
		**Fatigue**	Extreme tiredness resulting from mental or physical exertion or as a result of caring for the child. This can result in e.g. lack of energy or courage to cook a healthy meal.
	**Psychological**		
		**Anticipation**	Preparing oneself, looking ahead at things. E.g. cooking double portions to have food for multiple days.
		**Food knowledge**	Facts, information, acquaintance acquired through experience or education about food. E.g. people do not always know what to consider as 'healthy'.
		**Habits**	A settled or regular tendency or practice related to eating behavior, especially one that is hard to change or give up.
		**Planning**	The process of making plans or organizing oneself. E.g. making a planning with the partner about the groceries (when, what, where…).
		**Self-control**	The ability to control oneself, in particular one's emotions and desires, especially in difficult situations. E.g. by not buying any unhealthy foods.
		**Self-licensing**	Allowing oneself more; not being worried or compensating certain behaviors. E.g. people may feel it is okay to eat unhealthy because they engage in sport activities.
		**Weight-control**	Maintain/obtain a healthy body weight (postpartum).
	**Situational**		
		**Effort and convenience**	Presence or absence of a determined attempt related to eating behavior, the state of being (un)able to proceed with something without difficulty. This makes that some people e.g. do not feel like cooking or spending time on healthy meals, as it requires too much effort. This could thus result in the choice for more take away or unhealthy, convenient foods.
		**Practical and situational constraints**	Limitations and restrictions because of practical organizational or situational circumstances, such as unpredictable time schedules or work in the household that takes a lot of time.
		**Time constraints**	Time limitations or restrictions related to eating behavior. E.g. some people stated that they lack time to plan and prepare healthy meals.
		**Time opportunities**	Time limitations or restrictions related to eating behavior. E.g. some people stated that they lack time to plan and prepare healthy meals.
**Interpersonal**			
		**Adaptation to rhythm of baby**	The action or process of adapting oneself to the baby. E.g. time of dinner depends on the mood and needs of the baby at that moment.

	**Baby becomes priority**	The baby takes precedence over everything. E.g. some people stated that e.g. spending time or playing with the baby is a priority over cooking.
	**Baby needs attention**	All the attention goes to the baby instead of other, eating-related, tasks.
	**Dietary intake of baby**	Influence of daily intake of nutrients and food components that is given to the baby. E.g. the baby has to try and learn to eat different kinds of fruits and vegetables.
	**Practical constraints because of baby**	Limitations, restrictions or difficulties because of the baby. Some tasks, such as cooking or cleaning, are more challenging with the baby around.
	**Professional influence**	Advice and assistance from professionals, such as maternity help after birth.
	**Role model**	Mother or father showing a persuasive behavior to their new-born. E.g. some people explained that they want to be the good example for their baby, which includes eating healthy.
**Environmental**		
**Micro**		
	**Home food availability**	The amounts and types of food that are physically (un)available at home. E.g. when people don't buy (un)healthy foods.

Supplementary Table S3. Parent ratings of determinants (mean ±SD; rated on a scale from 0 to 10) and sex-differences (independent samples t- and p-value) during pregnancy.

**Determinants of changes in eating behavior during pregnancy**	**Women (n=57)** Mean ± SD	**Men (n=37)** Mean ± SD	t-value	p-value
**Level/sublevel**				
**Individual**				
**Biological**				
**Cravings**	4.7 ± 3.6	2.2 ± 2.8	3.490	**0.001**
**Specific food preferences**	4.7 ± 3.6	1.0 ± 2.1	6.391	**<0.001**
**Taste palette**	3.7 ± 3.3	0.8 ± 1.6	5.707	**<0.001**
**Taste preference**	4.7 ± 3.6	0.9 ± 2.1	6.434	**<0.001**
**Discomfort**	5.3 ± 3.6	0.8 ± 1.5	8.631	**<0.001**
**Fatigue**	5.7 ± 3.4	3.3 ± 3.2	3.434	**0.001**
**Hunger and satiety**	4.3 ± 3.2	1.5 ± 2.1	5.219	**<0.001**
**Physiological changes**	5.8 ± 3.2	/		
**Sickness**	5.1 ± 3.8	0.2 ± 0.6	9.469	**<0.001**
**Psychological**				
**(Perceived) food safety**	5.7 ± 3.6	2.7 ± 3.3	4.070	**<0.001**
**Food knowledge**	3.6 ± 3.7	3.2 ± 2.8	0.475	0.636
**Habits**	3.2 ± 2.8	2.2 ± 2.7	1.586	0.116
**Health consciousness**	4.9 ± 3.2	4.2 ± 3.1	1.015	0.313
**Health concerns**	7.3 ± 3.0	4.0 ± 3.4	5.019	**<0.001**
**Mood and emotions**	4.4 ± 3.5	3.2 ± 3.1	1.636	0.105
**Worries and concerns**	4.3 ± 3.6	2.1 ± 2.8	3.337	**0.001**

Pregnancy card, self-licensing	4.1 ± 3.4	2.6 ± 2.8	2.217	**0.029**
Anticipation	4.2 ± 3.3	1.9 ± 2.6	3.763	**<0.001**
Eating regulation	4.8 ± 3.1	3.0 ± 2.8	2.885	**0.005**
Self-control	4.1 ± 3.3	2.8 ± 2.6	1.958	0.053
Self-efficacy	3.1 ± 3.4	2.8 ± 2.6	1.628	0.107
Weight-control	5.2 ± 3.2	3.7 ± 2.9	2.245	**0.027**
**Situational**				
Effort and convenience	4.3 ± 3.1	2.2 ± 2.9	3.267	**0.002**
Time constraints	3.4 ± 3.1	2.5 ± 3.0	1.360	0.177
Other priorities	2.3 ± 2.5	2.6 ± 2.8	-0.576	0.566
**Interpersonal**				
Professional influence	4.7 ± 3.7	2.3 ± 3.1	3.311	**0.001**
Influence of male/female partner	3.4 ± 3.2	3.8 ± 2.8	-0.543	0.588
Sensitivity to others' opinion	3.0 ± 3.1	1.7 ± 2.3	2.419	**0.018**
Social pressure to eat	1.2 ± 1.8	1.3 ± 2.3	-0.221	0.825
**Environmental**				
**Micro**				
Home food availability	3.9 ± 3.5	3.6 ± 3.2	0.508	0.613
**Meso/Macro**				
Environment food availability	3.8 ± 3.3	2.4 ± 2.7	2.200	**0.030**

*Statistically significant p-values are highlighted in **bold***

Supplementary Table S4. Parent ratings of determinants (mean ± SD; rated on a scale from 0 to 10) and sex-differences (independent samples t- and p-value) postpartum.

Determinants of changes in eating behavior within the first year postpartum	Women (n=48) Mean ± SD	Men (n=37) Mean ± SD	t-value	p-value
**Level/sublevel**				
**Individual**				
**Biological**				
Discomfort	1.5 ± 2.3	0.8 ± 1.3	1.775	0.080
Fatigue	5.9 ± 2.9	5.2 ± 2.7	1.085	0.281
**Psychological**				
Food knowledge	2.8 ± 2.5	3.0 ± 2.9	-0.413	0.681
Habits	4.3 ± 3.2	3.9 ± 2.6	0.663	0.509
Self-licensing	4.5 ± 2.6	3.2 ± 2.8	2.135	**0.036**
Anticipation	5.0 ± 3.0	5.2 ± 3.0	-0.389	0.698
Planning	5.7 ± 2.7	5.0 ± 2.7	1.191	0.237
Self-control	4.5 ± 2.9	3.2 ± 2.8	2.108	**0.038**
Weight control	4.6 ± 3.0	3.1 ± 3.0	2.258	**0.027**
**Situational**				
Practical and situational constraints	5.3 ± 2.8	4.3 ± 2.5	1.758	0.082
Effort and convenience	5.0 ± 2.8	4.4 ± 2.8	1.015	0.313
Time constraints	5,3 ± 2.9	4.2 ± 2.5	1.988	0.050
Time opportunities	4.7 ± 2.8	4.0 ± 2.7	1.163	0.248

**Interpersonal**					
	**Professional influence**	2.3 ± 2.7	1.4 ± 2.0	1.757	0.083
	**Role model**	4.8 ± 3.3	3.7 ± 3.1	1.655	0.102
	**Dietary intake of baby**	6.2 ± 3.2	4.8 ± 3.4	2.011	**0.048**
	**Adaptation to rhythm of baby**	7.3 ± 2.2	5.8 ± 2.6	2.881	**0.005**
	**Baby becomes priority**	6.8 ± 2.7	5.1 ± 2.7	2.855	**0.005**
	**Baby needs attention**	6.4 ± 2.4	5.2 ± 2.6	2.301	**0.024**
	**Practical constraints because of baby**	6.6 ± 2.5	4.9 ± 2.5	3.065	**0.003**
**Environmental**					
**Micro**					
	**Home food availability**	4.6 ± 3.2	3.6 ± 3.2	1.496	0.138

Statistically significant p-values are highlighted in ***bold***

Supplementary Table S5. Expert ratings of determinants of changes in eating behavior during pregnancy.

**Determinants of changes in eating behavior during pregnancy**		**Modifiability**	**Relationship strength**	**Population-level effect**	**Priority for research score**	**N.A.***
		Mean ± SD	Mean ± SD	Mean ± SD	Mean ± SD	
**Level/sublevel**						
**Individual**						
**Biological**						
**Cravings**	Pregnant women	1.71 ± 0.66	1.46 ± 0.51	1.86 ± 0.80	1.92 ± 0.45	0/ 28
	Expecting father	1.18 ± 0.95	0.86 ± 0.65	0.96 ± 0.79	1.14 ± 0.81	8/ 27
**Specific food preferences**	Pregnant women	1.48 ± 0.70	1.56 ± 0.51	1.70 ± 0.82	1.84 ± 0.52	0/ 27
	Expecting father	1.04 ± 0.94	1.00 ± 0.78	0.85 ± 0.66	1.13 ± 0.84	8/ 27
**Taste palette**	Pregnant women	1.57 ± 0.74	1.36 ± 0.49	1.57 ± 0.69	1.73 ± 0.47	0/ 28
	Expecting father	0.68 ± 0.77	0.57 ± 0.57	0.64 ± 0.73	0.73 ± 0.74	13/ 28
**Taste preference**	Pregnant women	1.37 ± 0.63	1.37 ± 0.49	1.52 ± 0.70	1.65 ± 0.46	0/ 27
	Expecting father	0.78 ± 0.75	0.78 ± 0.70	0.78 ± 0.70	0.91 ± 0.77	10/ 27
**Discomfort**	Pregnant women	1.61 ± 0.74	1.54 ± 0.51	1.75 ± 0.80	1.89 ± 0.63	0/ 28
	Expecting father	0.71 ± 0.76	0.78 ± 0.80	0.81 ± 0.96	0.90 ± 0.90	12/ 27
**Fatigue**	Pregnant women	1.50 ± 0.69	1.43 ± 0.50	1.64 ± 0.68	1.76 ± 0.46	0/ 28
	Expecting father	1.25 ± 1.01	0.93 ± 0.66	1.00 ± 0.72	1.21 ± 0.82	7/ 28
**Hunger and satiety**	Pregnant women	2.00 ± 0.73	1.44 ± 0.51	1.93 ± 0.78	2.03 ± 0.59	0/ 27
	Expecting father	1.67 ± 0.83	1.26 ± 0.66	1.48 ± 0.89	1.67 ± 0.77	3/ 24
**Physiological changes**	Pregnant women	1.41 ± 0.50	1.37 ± 0.49	1.48 ± 0.64	1.65 ± 0.46	0/ 27
**Sickness**	Pregnant women	1.45 ± 0.69	1.59 ± 0.50	1.79 ± 0.77	1.87 ± 0.43	0/ 29
	Expecting father	0.55 ± 0.78	0.41 ± 0.50	0.48 ± 0.63	0.55 ± 0.69	17/ 29
**Psychological**						
**Perceived food safety**	Pregnant women	2.11 ± 0.69	1.39 ± 0.50	1.82 ± 0.77	2.01 ± 0.53	0/ 28
	Expecting father	1.00 ± 1.05	0.64 ± 0.62	0.82 ± 0.86	0.93 ± 0.89	12/ 28
**Food knowledge**	Pregnant women	2.73 ± 0.52	1.53 ± 0.51	2.20 ± 0.76	2.41 ± 0.47	0/ 30
	Expecting father	2.37 ± 0.85	1.43 ± 0.57	1.87 ± 0.78	2.13 ± 0.65	1/ 30
**Habits**	Pregnant women	2.30 ± 0.67	1.63 ± 0.49	2.15 ± 0.72	2.30 ± 0.54	0/ 27
	Expecting father	2.07 ± 0.83	1.48 ± 0.58	1.93 ± 0.78	2.07 ± 0.70	1/ 27
	Pregnant women	2.41 ± 0.69	1.50 ± 0.51	2.22 ± 0.80	2.31 ± 0.59	0/ 26

**Health consciousness**	Expecting father	2.11 ± 0.85	1.44 ± 0.58	2.00 ± 0.88	2.09 ± 0.73	1/ 27
**Health concerns**	Pregnant women	2.50 ± 0.63	1.60 ± 0.50	2.27 ± 0.74	2.39 ± 0.53	0/ 30
	Expecting father	1.83 ± 0.79	1.33 ± 0.55	1.80 ± 0.81	1.88 ± 0.63	1/ 30
**Mood and emotions**	Pregnant women	1.77 ± 0.59	1.36 ± 0.49	1.81 ± 0.75	1.88 ± 0.53	0/ 25
	Expecting father	1.64 ± 0.64	1.32 ± 0.56	1.50 ± 0.71	1.72 ± 0.62	1/ 24
**Worries and concerns**	Pregnant women	2.22 ± 0.64	1.33 ± 0.48	1.89 ± 0.70	2.04 ± 0.51	0/ 27
	Expecting father	1.67 ± 0.88	1.07 ± 0.55	1.41 ± 0.75	1.56 ± 0.73	3/ 27
**Pregnancy card. self licensing**	Pregnant women	2.07 ± 0.70	1.38 ± 0.56	1.72 ± 0.70	1.95 ± 0.61	1/ 29
	Expecting father	1.52 ± 1.06	1.04 ± 0.69	1.24 ± 0.83	1.45 ± 0.89	6/ 28
**Anticipation**	Pregnant women	2.30 ± 0.78	1.41 ± 0.57	1.93 ± 0.87	2.11 ± 0.70	1/ 27
	Expecting father	2.11 ± 0.89	1.33 ± 0.62	1.67 ± 0.88	1.93 ± 0.78	2/ 27
**Eating regulation**	Pregnant women	2.03 ± 0.49	1.47 ± 0.51	2.03 ± 0.78	2.10 ± 0.53	0/ 29
	Expecting father	1.83 ± 0.70	1.30 ± 0.54	1.69 ± 0.76	1.83 ± 0.63	1/ 29
**Self-control**	Pregnant women	1.96 ± 0.59	1.48 ± 0.51	2.04 ± 0.76	2.07 ± 0.47	0/ 27
	Expecting father	1.67 ± 0.83	1.26 ± 0.66	1.63 ± 0.97	1.73 ± 0.81	3/ 27
**Self-efficacy**	Pregnant women	2.18 ± 0.72	1.64 ± 0.49	1.96 ± 0.79	2.20 ± 0.52	0/ 28
	Expecting father	1.96 ± 0.96	1.48 ± 0.64	1.71 ± 0.90	1.96 ± 0.79	2/ 27
**Weight-control**	Pregnant women	2.36 ± 0.56	1.64 ± 0.49	2.32 ± 0.61	2.09 ± 0.45	0/ 28
	Expecting father	2.00 ± 0.94	1.29 ± 0.66	1.71 ± 0.90	1.88 ± 0.84	3/ 28
**Situational**						
**Effort and convenience**	Pregnant women	2.07 ± 0.60	1.32 ± 0.48	1.78 ± 0.75	1.95 ± 0.50	0/ 27
	Expecting father	1.89 ± 0.74	1.29 ± 0.53	1.56 ± 0.75	1.80 ± 0.59	1/ 27
**Time constraints**	Pregnant women	1.97 ± 0.57	1.36 ± 0.49	1.86 ± 0.59	1.95 ± 0.52	0/ 28
	Expecting father	1.82 ± 0.77	1.22 ± 0.58	1.74 ± 0.76	1.80 ± 0.73	2/ 27
**Other priorities**	Pregnant women	2.00 ± 0.54	1.21 ± 0.42	1.68 ± 0.61	1.83 ± 0.46	0/ 28
	Expecting father	1.71 ± 0.81	1.04 ± 0.51	1.39 ± 0.74	1.55 ± 0.67	3/ 28
**Interpersonal**						
**Professional influence**	Pregnant women	2.66 ± 0.55	1.52 ± 0.51	2.34 ± 0.67	2.43 ± 0.40	0/ 29
	Expecting father	1.72 ± 1.16	1.11 ± 0.74	1.69 ± 1.07	1.71 ± 0.99	5/ 28
**Influence of partner**	Pregnant women	2.11 ± 0.80	1.44 ± 0.64	1.81 ± 0.79	2.03 ± 0.65	0/ 27
	Expecting father	2.18 ± 0.72	1.29 ± 0.54	1.50 ± 0.69	1.87 ± 0.59	1/ 28
**Sensitivity to other opinions**	Pregnant women	1.96 ± 0.64	1.22 ± 0.42	1.61 ± 0.74	1.78 ± 0.40	0/ 27
	Expecting father	1.32 ± 0.86	0.96 ± 0.58	1.11 ± 0.79	1.29 ± 0.70	5/ 28
**Socail pressure to eat**	Pregnant women	2.15 ± 0.53	1.44 ± 0.51	1.81 ± 0.74	2.04 ± 0.48	0/ 27
	Expecting father	1.22 ± 1.09	0.85 ± 0.77	0.89 ± 0.93	1.13 ± 1.00	10/ 27
**Environmental**						
**Micro**						
**Home food availability**	Pregnant women	2.44 ± 0.64	1.41 ± 0.50	2.15 ± 0.72	2.23 ± 0.58	0/ 27
	Expecting father	2.33 ± 0.78	1.33 ± 0.56	1.96 ± 0.85	2.10 ± 0.71	1/ 27
**Meso/Macro**						
**Environment food availability**	Pregnant women	1.89 ± 0.75	1.38 ± 0.50	1.96 ± 0.71	1.97 ± 0.60	0/ 26
	Expecting father	1.54 ± 1.03	1.08 ± 0.69	1.52 ± 0.98	1.58 ± 0.94	5/ 25

**Number of experts indicating NA relative to the total number of experts rating the determinant*

Priority for research-score per determinant was calculated by using the following formula: (mean score on modifiability/3 + mean score on relationship strength/2 + mean score on population-level effect/3).

Supplementary Table S6. Expert ratings of determinants of changes in eating behavior postpartum.

Determinants of changes in eating behavior within the first year postpartum		Modifiability	Relationship strength	Population-level effect	Priority for research score	N.A.*
		Mean ± SD	Mean ± SD	Mean ± SD	Mean ± SD	
**Level/sublevel**						
**Individual**						
**Biological**						
**Fatigue**	First-time mother	1.42 ± 0.64	1.54 ± 0.51	1.96 ± 0.87	1.90 ± 0.45	0/ 26
	First-time father	1.35 ± 0.56	1.42 ± 0.58	1.69 ± 0.88	1.72 ± 0.58	1/ 26
**Discomfort**	First-time mother	1.27 ± 0.45	1.23 ± 0.43	1.31 ± 0.55	1.47 ± 0.34	0/ 26
	First-time father	0.96 ± 0.66	0.96 ± 0.66	0.92 ± 0.65	1.10 ± 0.71	6/ 24
**Psychological**						
**Food knowledge**	First-time mother	2.62 ± 0.57	1.50 ± 0.51	2.00 ± 0.69	2.29 ± 0.53	0/ 26
	First-time father	2.54 ± 0.65	1.46 ± 0.51	1.88 ± 0.65	2.21 ± 0.54	0/ 26
**Habits**	First-time mother	2.04 ± 0.66	1.58 ± 0.50	1.81 ± 0.63	2.07 ± 0.44	0/ 26
	First-time father	1.88 ± 0.82	1.54 ± 0.58	1.73 ± 0.72	1.97 ± 0.62	1/ 26
**Self-licensing**	First-time mother	2.04 ± 0.66	1.38 ± 0.50	1.58 ± 0.64	1.90 ± 0.51	0/ 26
	First-time father	1.92 ± 0.74	1.31 ± 0.47	1.50 ± 0.65	1.79 ± 0.50	0/ 26
**Anticipation**	First-time mother	2.23 ± 0.71	1.42 ± 0.50	1.96 ± 0.66	2.11 ± 0.58	0/ 26
	First-time father	2.12 ± 0.82	1.38 ± 0.57	1.81 ± 0.69	2.00 ± 0.68	1/ 26
**Planning**	First-time mother	2.38 ± 0.80	1.27 ± 0.53	1.31 ± 0.47	2.03 ± 0.67	1/ 26
	First-time father	2.31 ± 0.79	1.31 ± 0.47	1.81 ± 0.85	2.01 ± 0.59	0/ 26
**Self-control**	First-time mother	1.88 ± 0.52	1.42 ± 0.50	1.69 ± 0.62	1.90 ± 0.41	0/ 26
	First-time father	1.81 ± 0.57	1.38 ± 0.50	1.62 ± 0.64	1.83 ± 0.46	0/ 26
**Weight control**	First-time mother	2.35 ± 0.69	1.62 ± 0.50	2.04 ± 0.77	2.27 ± 0.54	0/ 26
	First-time father	1.88 ± 0.95	1.42 ± 0.64	1.62 ± 0.90	1.88 ± 0.82	2/ 26
**Situational**						
**Practical and situational constraints**	First-time mother	1.65 ± 0.63	1.42 ± 0.50	1.81 ± 0.69	1.87 ± 0.52	0/ 26
	First-time father	1.50 ± 0.65	1.31 ± 0.47	1.62 ± 0.64	1.69 ± 0.53	0/ 26
**Effort and convenience**	First-time mother	2.04 ± 0.72	1.60 ± 0.50	1.81 ± 0.75	2.09 ± 0.56	0/ 25
	First-time father	1.88 ± 0.71	1.50 ± 0.51	1.76 ± 0.66	1.99 ± 0.53	0/ 25
**Time constraints**	First-time mother	1.62 ± 0.64	1.35 ± 0.49	1.81 ± 0.69	1.81 ± 0.49	0/ 26
	First-time father	1.58 ± 0.58	1.35 ± 0.49	1.69 ± 0.62	1.76 ± 0.45	0/ 26
**Time opportunities**	First-time mother	1.96 ± 0.79	1.44 ± 0.51	2.08 ± 0.70	2.07 ± 0.55	0/ 25
	First-time father	1.84 ± 0.75	1.40 ± 0.50	1.84 ± 0.75	1.93 ± 0.63	0/ 25
**Interpersonal**						
**Professional influence**	First-time mother	2.56 ± 0.58	1.48 ± 0.51	2.12 ± 0.60	2.31 ± 0.47	0/ 25
	First-time father	2.35 ± 0.94	1.41 ± 0.64	1.68 ± 0.85	2.07 ± 0.79	2/ 24
**Role model**	First-time mother	2.27 ± 0.67	1.46 ± 0.51	2.00 ± 0.82	2.18 ± 0.54	0/ 25
	First-time father	2.04 ± 0.82	1.42 ± 0.58	1.84 ± 0.85	2.03 ± 0.69	1/ 25
**Dietary intake of baby**	First-time mother	2.31 ± 0.74	1.38 ± 0.50	1.81 ± 0.80	2.06 ± 0.60	0/ 26
	First-time father	2.19 ± 0.80	1.38 ± 0.50	1.77 ± 0.77	2.01 ± 0.61	0/ 26
**Adaptation to rhythm baby**	First-time mother	1.60 ± 0.71	1.32 ± 0.48	1.88 ± 0.78	1.82 ± 0.53	0/ 25
	First-time father	1.44 ± 0.51	1.32 ± 0.48	1.64 ± 0.64	1.69 ± 0.44	0/ 25
	First-time mother	1.69 ± 0.68	1.27 ± 0.45	1.77 ± 0.65	1.79 ± 0.43	0/ 26

		**Baby becomes priority**	First-time father	1.65 ± 0.63	1.23 ± 0.43	1.69 ± 0.62	1.73 ± 0.45	0/ 26
		**Baby needs attention**	First-time mother	1.62 ± 0.64	1.19 ± 0.40	1.85 ± 0.78	1.75 ± 0.46	0/ 26
			First-time father	1.50 ± 0.51	1.19 ± 0.40	1.54 ± 0.76	1.61 ± 0.45	0/ 26
		**Practical and situational constraints because of baby**	First-time mother	1.65 ± 0.63	1.35 ± 0.49	1.96 ± 0.77	1.88 ± 0.48	0/ 26
			First-time father	1.50 ± 0.51	1.35 ± 0.49	1.80 ± 0.71	1.79 ± 0.45	0/ 25
**Environmental**								
	**Micro**							
		**Home food availability**	First-time mother	2.50 ± 0.65	1.58 ± 0.50	2.19 ± 0.80	2.35 ± 0.55	0/ 26
			First-time father	2.46 ± 0.65	1.58 ± 0.50	2.19 ± 0.80	2.34 ± 0.56	0/ 26

**Number of experts indicating NA relative to the total number of experts rating the determinant*

Priority for research-score per determinant was calculated by using the following formula: (mean score on modifiability/3 + mean score on relationship strength/2 + mean score on population-level effect/3).
